# Pulmonary vein volume predicts the outcome of radiofrequency catheter ablation of paroxysmal atrial fibrillation

**DOI:** 10.1371/journal.pone.0201199

**Published:** 2018-07-25

**Authors:** Keiko Shimamoto, Fumiharu Miura, Yuji Shimatani, Kenji Nishioka, Ichiro Inoue

**Affiliations:** Department of Cardiology, Hiroshima City Hospital, Hiroshima City, Hiroshima, Japan; Indiana University, UNITED STATES

## Abstract

**Purpose:**

Catheter ablation of atrial fibrillation (AF) is an effective therapy for selected groups of patients. We evaluated whether quantification of left atrium (LA) or pulmonary vein (PV) by using multi-detector computed tomography (MDCT) may predict the success rate of PV isolation procedure.

**Methods:**

We included 118 patients younger than 65 years with symptomatic AF (73 paroxysmal, PAF; 45 non-paroxysmal, non-PAF). All patients underwent 256-slice MDCT prior to circumferential PV isolation to evaluate anatomy, volume and dimensions of LA and PV.

**Results:**

After a mean follow-up of 14 months, complete success was achieved in 50 patients (68.5%) of PAF and in 26 patients (57.8%) of non-PAF. In the PAF group, total PV volume was found to be an independent predictor of AF recurrence, whereas LA volume was not. Logistic regression analysis showed that the probability of AF recurrence was higher in patients with total PV volume greater than 12.0 cm^3^/BSA (m^2^) (AUC 0.682, 95%CI 0.541―0.822). In the non-PAF group, no independent risk factor of LA or PV size was observed for the postoperative recurrence.

**Conclusions:**

The PV volume quantification may predict the success of AF ablation in PAF patients.

## Introduction

Radiofrequency catheter ablation (RFCA) of atrial fibrillation (AF) is becoming an effective therapy for selected groups of patients, whose success rate may depend on patient characteristics. Previous studies identified large left atrial (LA) and pulmonary vein (PV) size as risk factors for early recurrence of AF after RFCA, which also included older age, hypertension, presence of multiple AF foci and duration of AF prior to RFCA [1,2]. Pulmonary veins (PVs), in particular the sleeves of atrial myocardium extending into the PVs, have been recognized as a major source of ectopic arrhythmogenic foci initiating AF [3]. Thus, PV isolation and circumferential pulmonary ablation are the most commonly used in RFCA procedures. LA size is also an important risk factor for AF, postablation recurrence, and progression of paroxysmal to persistent AF. Multi-detector computed tomography (MDCT) allows accurate assessment of anatomy of LA and PV, including the PV ostia, to investigate the preprocedural predictors of AF recurrence after RFCA. Furthermore, the prevalence of AF in the elderly, especially those over 65 years old, was higher than that in younger adults. To minimize the influence of aging as a risk factor of AF, we selected patients younger than 65 years without structural heart disease. In this study, we evaluated whether quantification of LA or PV by using MDCT may predict the success rate of PV isolation procedure, giving us the information for AF patient prognosis and selection for ablation.

## Methods

### Patients

The study population included 118 consecutive patients (102 males, mean age 53.3 ± 8.1 years, range 30 to 64 years) who underwent their first AF ablation procedure at Hiroshima City Hospital, Japan between April 2010 and March 2013. Written informed consent was obtained from all patients. Demographic, clinical, MDCT imaging, and echocardiographic data were prospectively entered in the department of cardiology information system and retrospectively analyzed. The Institutional Review Board of the Hiroshima City Hospital approved the retrospective analysis of clinically acquired data. Exclusion criteria were severe mitral regurgitation or stenosis, previous mitral surgery, atrial flutter, congenital heart disease, hypertrophic or dilated cardiomyopathy, acute myocardial infarction, left ventricular dysfunction (left ventricular ejection fraction < 50%), chronic kidney disease (eGFR < 45 ml/min/1.73 m^2^), hyperthyroidism, and chronic obstructive pulmonary disease. The patients with a patent foramen ovale or those with transient LV dysfunction primarily induced by AF tachycardia were not excluded.

All patients underwent a transesophageal echocardiography 24―72 h before RFCA to exclude the presence of thrombi. Patients were classified into the paroxysmal AF (PAF, n = 73) and the non-paroxysmal AF (non-PAF, n = 45) groups, according to the American College of Cardiology/American Heart Association/European Society of Cardiology guidelines. PAF was defined as recurrent AF (≥2 episodes) that terminates spontaneously within 7 days. Non-PAF was defined as continuous AF sustained beyond 7 days at least once. All non-PAF patients previously pursue rhythm control therapy. Considering that clear division between persistent and longstanding persistent AF patients was difficult because 80% of patients had few symptoms of AF, longstanding persistent AF patients might have been included in the non-PAF group. None of the patients had specific ECG, clinical episodes of syncope or aborted cardiac arrest, or family histories that strongly indicate hereditary arrhythmia syndrome. Lack of genetic testing could not rule out the possibility that AF patients aged ≤40 years might have some genetic background that cause AF. Patients’ demographics are shown in Table 1.

**Table 1:** Clinical characteristics of patients in the PAF and non-PAF groups.

Variables	Total	PAF	non-PAF	P Value
Patients, n (%)	118	73 (61.8)	45 (38.1)	
Male sex, n (%)	102(86.4)	61 (83.5)	41 (91.1)	0.28
Age, years (IQR)	58 (50–62)	59 (49.5–62)	58 (51–62)	0.98
BMI, kg/m^2^ (IQR)	23.8 (21.6–26.5)	23.8 (21.7–26.7)	23.9(21.3–26.1)	0.88
Duration of AF, months (IQR)	26 (7–72)	36(6.5–72)	24 (7–14)	0.46
Hypertension, n (%)	37 (31.3)	23 (31.5)	14 (31.1)	1
Diabetes mellitus, n (%)	11(9.3)	7(9.6)	4(8.9)	1
Stroke, n (%)	10(8.5)	5(6.8)	5(11.1)	0.50
History of TCM, n (%)	9(7.6)	5(6.8)	4(8.9)	0.73
Pre-ablation anti-arrhythmic drugs				
Class Ⅰ AAD, n (%)	46(39.0)	37(50.7)	9(20.0)	<0.001*
Class Ⅲ AAD, n (%)	2(1.7)	1(1.4)	1(2.2)	1
βblocker, n (%)	50(42.4)	27(37.0)	23(51.1)	0.18
ACEI/ARB, n (%)	34(28.8)	18(24.7)	16(35.6)	0.22
Statin, n (%)	25(21.2)	12(16.4)	13(28.9)	0.16
LV ejection fraction, % (IQR)	67(61.8–71.0)	69(65–72)	64(59–68)	<0.0001*
Left atrial anteroposterior echocardiographic dimension, mm (IQR)	41(37–44)	40(36–43)	42(39–46)	0.008*
NT proBNP, pg/ml (IQR)	118(51–382)	67(30–135)	391(223–818)	<0.0001*
Recurrent patients, n (%)	42(35.6)	23(31.5)	19(42.2)	0.23
Recurrence time, months (IQR)	6.5(3.2–9.7)	7.0(4.0–10.5)	6.0(3.0–9.0)	0.75

Data are n (%), and median (IQR; 25%―75% interquartile range).

* significant difference

AAD, anti-arrhythmic drug; ACEI, angiotensin converting enzyme inhibitor; ARB, angiotensin II receptor blocker; BMI, body mass index; LV, left ventricle; non-PAF, non-paroxysmal atrial fibrillation; PAF, paroxysmal atrial fibrillation; TCM, tachycardia-induced cardiomyopathy

During post-ablation period, no significant difference in antiarrhythmic treatment was found between PAF and non-PAF patients (Table 2).

**Table 2:** Pharmacological antiarrhythmic treatment pre- and post-ablation.

	pre-ablation			post-ablation		
	Total	PAF	non-PAF	Total	PAF	non-PAF
Patients, n	118	73	45	118	73	45
**Class Ia**						
cibenzoline	13 (11.0)	10 (13.7)	3 (6.7)	2 (1.7)	1 (1.4)	1 (2.2)
disopyramide	4 (3.4)	4 (5.5)	0 (0.0)	1 (0.9)	1 (1.4)	0 (0.0)
pirmenol	1 (0.9)	1 (1.4)	0 (0.0)	0 (0.0)	0 (0.0)	0 (0.0)
**Class Ib**						
aprindine	2 (1.7)	1 (1.4)	1 (2.2)	2 (1.7)	1 (1.4)	1 (2.2)
**Class Ic**						
pilsicainide	18 (15.3)	16 (21.9)	2 (4.4)	5 (4.2)	5 (6.8)	0 (0.0)
flecainide	8 (6.8)	5 (6.8)	3 (6.7)	8 (6.8)	6 (8.2)	2 (4.4)
propafenone	1 (0.9)	1 (1.4)	0 (0.0)	0 (0.0)	0 (0.0)	0 (0.0)
**Class III**						
amiodarone	2 (1.7)	1 (1.4)	1 (2.2)	1 (0.9)	1 (1.4)	0 (0.0)
sotalol	0 (0.0)	0 (0.0)	0 (0.0)	2 (1.7)	2 (2.7)	0 (0.0)
**β blocker**						
bisoprolol	31 (26.3)	16 (21.9)	15 (33.3)	16 (13.6)	10 (13.7)	6 (13.3)
carvedilol	14 (11.9)	10 (13.7)	4 (8.9)	5 (4.2)	4 (5.5)	1 (2.2)
atenolol	6 (5.1)	3 (4.1)	3 (6.7)	2 (1.7)	0 (0.0)	2 (4.4)
**Calcium channel blocker**						
bepridil	25 (21.2)	11 (15.1)	14 (31.1)	13 (11.0)	7 (9.6)	6 (13.3)
verapamil	4 (3.4)	3 (4.1)	1 (2.2)	1 (0.9)	0 (0.0)	1 (2.2)
digoxin	6 (5.1)	3 (4.1)	3 (6.7)	0 (0.0)	0 (0.0)	0 (0.0)

Data are n (%). non-PAF, non-paroxysmal atrial fibrillation; PAF, paroxysmal atrial fibrillation

### Contrast enhanced electrocardiogram-gated MDCT

MDCT scan was performed systematically up to 3 days before the procedure, for electroanatomic mapping integration, PV anatomy delineation, LA thrombus exclusion, and LA and PV volume estimation, using a 256-slice helical-scanning system (Brilliance iCT, Philips Healthcare, Cleveland, OH, USA). Scanning was performed in a single breath hold in the cranio-caudal direction from the aortic arch to the diaphragm, being gated to the cardiac cycle through electrocardiogram synchronization. Nonionic iodine contrast medium (I, 24 mg /kg/s) was injected at a flow-rate of 4―5 ml/s.

### RFCA and follow-up

Extensive encircling PV isolation was performed. All patients received intravenous heparin to maintain an activated clotting time of 300―400 s. Intracardiac echocardiography was used to exclude a cardiac thrombus and to guide the trans-septal puncture. A non-fluoroscopic electroanatomical mapping system with multislice CT integration was employed to guide either ablation procedure: CARTO (Biosense–Webster Inc., Diamond Bar, CA, USA) or EnSite NavX™ (St. Jude Medical, Inc., Minneapolis, MN, USA). The ipsilateral PVs were encompassed as a single unit using a 3.5-mm irrigated-tip ablation catheter with a maximal temperature of 40°C and a maximal power of 25―30W until a bipolar voltage of < 0.1 mV was achieved. Conduction block was subsequently verified by repeated administration of 20 mg adenosine after isoproterenol injection during sinus rhythm or pacing in the coronary sinus. If AF was sustained after PV isolation, additional ablation consisting of superior vena cava isolation, creating LA linear lesion (roof, posterior wall, or mitral isthmus), ablation of complex atrial fractionated electrograms and/or ganglionated plexi ablation was performed. We did not induced AF during electrophysiological procedures in a fixed protocol. In addition to the standard AF ablation, the cavotricuspid isthmus conduction block was confirmed in all cases.

Patients were routinely followed-up at the outpatient clinic 1, 3, and 6 months after the procedures. Clinical success was assessed after a 3-month blanking period. It was defined as the absence of symptomatic or documented AF by periodic electrocardiograms and 24-hour Holter monitoring. Anti-arrhythmic drug therapy was discontinued or modified at the clinician’s decision in periodic evaluations.

### Measurements

**Fig 1:**
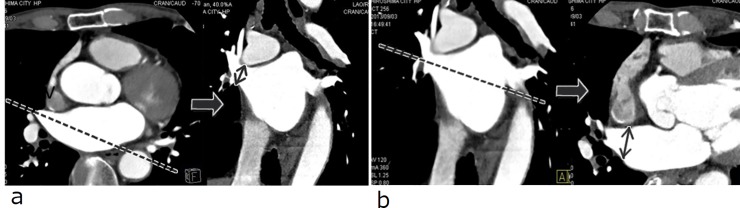
Identification and labeling of the right superior pulmonary vein (RSPV) ostia in two-dimensional viewing. (a) Measurement of superoinferior diameter of RSPV ostium (solid arrow). (b) Measurement of anteroposterior diameter of RSPV ostium (solid arrow).

LA and PVs were reconstructed from MDCT data using a specialized software (Syngo InSpace EP, Siemens). To determine the long axis of the PV, axial and coronal tomograms at the junction of the PV and LA were displayed, and then oblique coronal and oblique axial images were identified parallel to each PV. The PV ostium was defined as the point of inflection between the LA wall and the PV wall or as the line crossing the carina perpendicular to the long axis of the PV. The ostium of the right superior PV was difficult to detect because of its funnel shape. To overcome this obstacle, multiple oblique coronal planes were used to detect the upper wall of the right inferior PV as a marker of the border between the LA and PV. The ostial superoinferior and anteroposterior lengths of PVs were measured (Fig 1), and the PV ostial areas were calculated using the ellipsoid formula.

**Fig 2:**
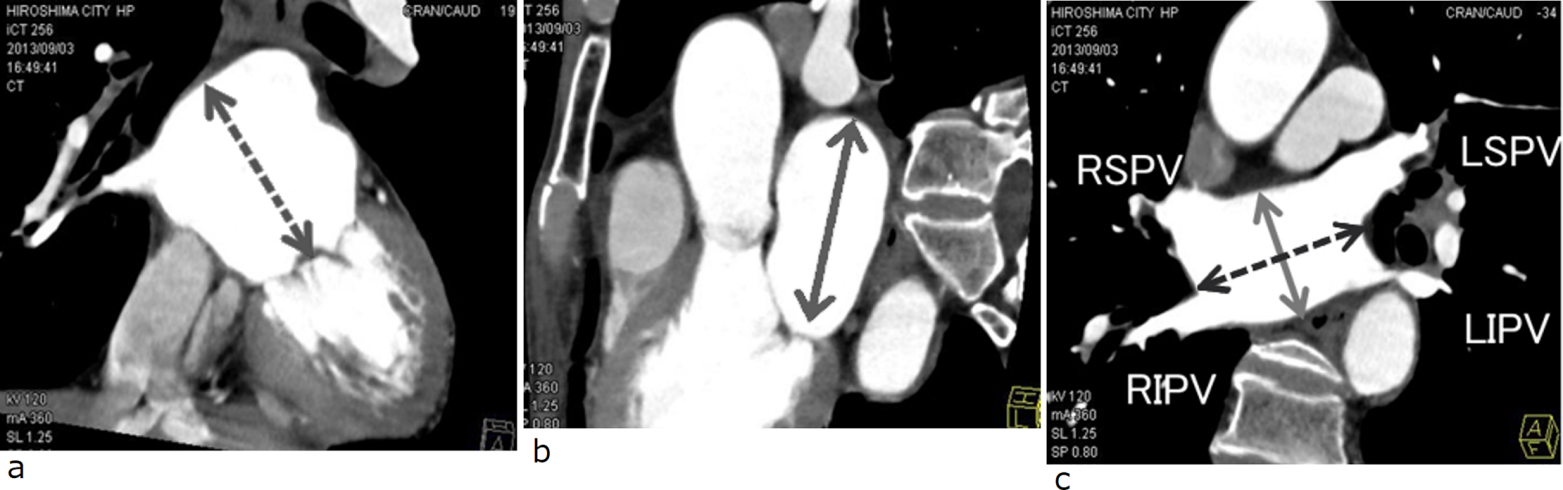
Identification of labeling of the left atrium (LA) in two-dimensional viewing. (a) LA top―mitral annulus diameter (dashed arrow). (b) LA longitudinal diameter (solid arrow). (c) LA anteroposterior diameter (solid arrow) and LA transverse diameter (dashed arrow).

The size of the LA was measured according to the technique used by Ho et al [4] (Fig 2). The measurement of transverse diameter of the LA was defined as the distance between the midpoint of the right and left sides of the PVs in oblique axial images. The anteroposterior and longitudinal diameters were measured at the midpoint of the transverse diameter in oblique axial and sagittal images. LA top―mitral annulus diameter was determined as the length between the level of the mitral annulus and the roof of the LA. Dimensions were measured to the nearest millimeter.

**Fig 3:**
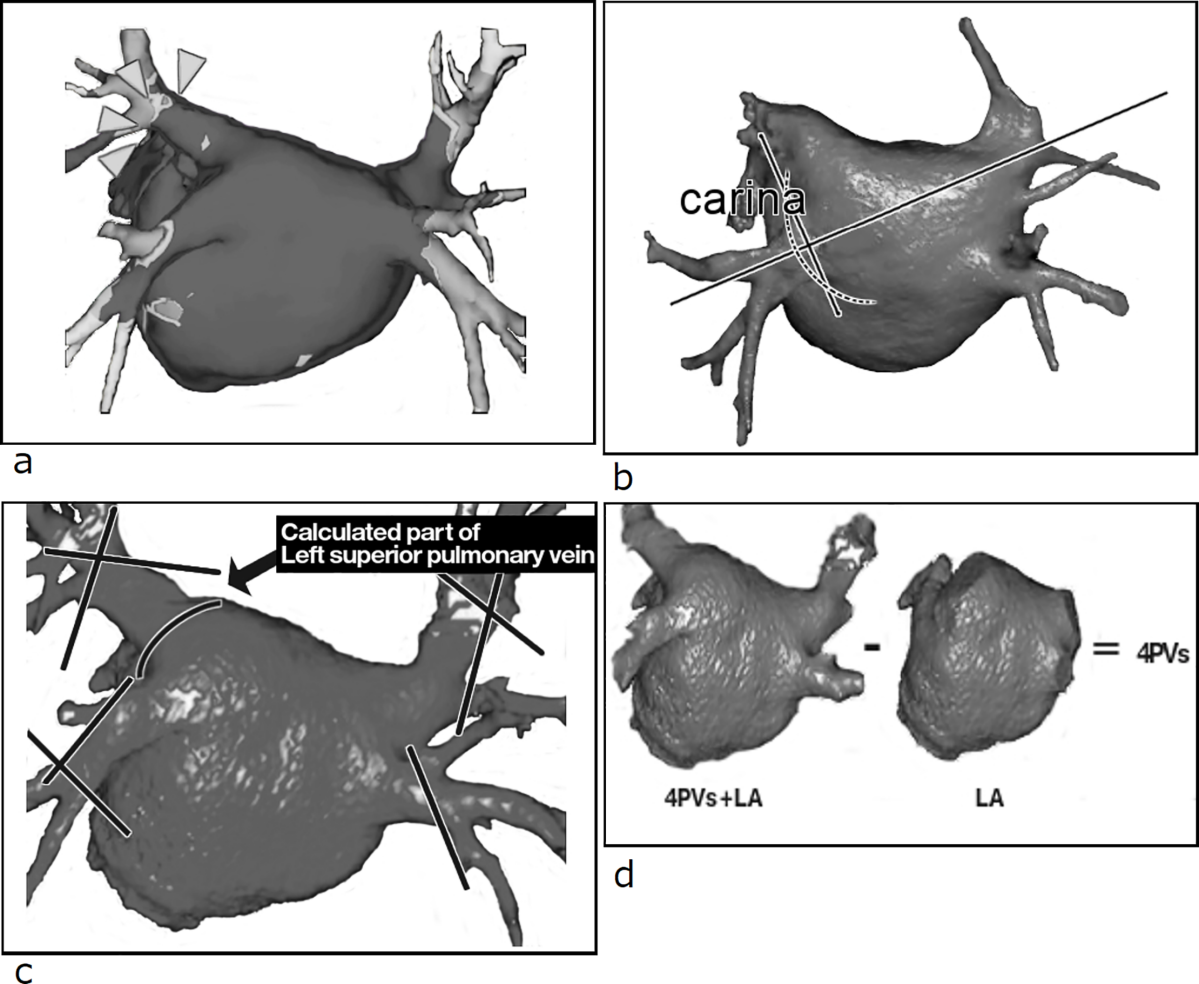
Three-dimensional volume-rendered reconstruction of the left atrium (LA) and pulmonary vein (PV). (a) Definition of the PV distal border as the first major branch in the simultaneously acquired voltage map (arrow head). (b) Definition of the PV―LA junction (dashed line). (c) Definition of the PV distal border and PV―LA junction. (d) Measurement of total PV volume and LA volume.

Three-dimensional-volume rendering was used to measure LA and PV volumes (Fig 3). PV volume was measured as the summation of four PV volumes between the PV―LA junction and the PV distal border. To determine the PV distal border, we used the simultaneously acquired voltage mapping obtained during RFCA with a non-fluoroscopic electroanatomical mapping system with multislice CT integration. This PV distal border was identical with the point of the major first bifurcation. Inside the main trunk of the PV, the main activation was located just before the first major branch point (Fig 3A). The PV―LA junction was defined as the perpendicular plane to the longitudinal axis containing both points of each PV carina in the typical bifurcation branching pattern (Fig 3B and 3C). The LA volume was subtracted from the total LA and PV volumes to thereby obtain four PV volumes (Fig 3D). In anatomical variation cases with the common left PV trunk, the trunk portion was included in the PV volume. The accessory veins were not counted in the PV volume. PV and LA volumes were indexed to body surface area.

The branching pattern of the PV anatomy was classified on the basis of the description of Kato et al [5]. No significant difference in PV anatomical variation was found between the PAF and non-PAF groups (Table 3).

**Table 3:** Anatomical variation of PV in the PAF and non-PAF groups.

	Total	PAF	non-PAF
Patients, n	118	73	45
Left common trunk			
Total	29 (24.5)	17 (23.2)	12 (24.4)
Long left common trunk > 1 cm	3 (2.5)	2 (2.7)	1 (2.2)
Accessary vein			
Right middle PV	28 (23.7)	15 (20.5)	13 (28.9)
Left middle PV	1 (0.9)	1 (1.4)	0 (0.0)
Upper PV	2 (1.7)	1 (1.4)	1 (2.2)

Data are n (%). non-PAF, non-paroxysmal atrial fibrillation; PAF, paroxysmal atrial fibrillation; PV, pulmonary vein

The upper PV was an anomalous vein distinct from the superior PV.

### Statistics

Data are given as median (IQR, 25%―75% interquartile range) and mean ± standard deviation. Statistical analysis was performed using EZR (Saitama Medical Center, Jichi Medical University, Saitama, Japan), which is a graphical user interface for R (The R Foundation for Statistical Computing, Vienna, Austria). More precisely, it is a modified version of R commander designed to add statistical functions frequently used in biostatistics [6]. One-way analysis of variance, Wilcoxon signed-rank test, and Tukey’s honestly significant difference test were used to compare variables, as appropriate. Categorical variables were compared with the χ^2^-test or Fisher’s exact test when appropriate. Multiple logistic regression analysis was performed to determine the predictors of recurrence after RFCA. A receiver-operating characteristic (ROC) curve was plotted for each predictor, and the area under the curve was calculated as an index for the predictive volume of the model. P < 0.05 was considered to be statistically significant.

## Results

Complete PV electrical isolation was obtained in all 118 patients submitted to their first RFCA. Cavo-tricuspid isthmus ablation was performed in 67 of 73 patients in the PAF group and in all 45 patients in the non-PAF group. At the end of the mean follow-up period of 14 months, 50 patients (68.5%) of the PAF group and 26 patients (57.8%) of the non-PAF group remained free from AF recurrence. No difference was found in the recurrence rate between the PAF and non-PAF groups. AF recurrence occurred mainly at the 7th and 6th months of follow-up in the PAF and non-PAF groups, respectively (Table 1). In the PAF group, patients without recurrence had greater body mass index compared with recurrent patients. In the non-PAF group, recurrent patients showed longer duration of AF than non-recurrent patients did. No differences in sex, age, co-morbidity, medication, or echocardiographic findings were observed between those with and without recurrence of AF in the PAF group, as well as in the non-PAF group (Table 4).

**Table 4:** Clinical characteristics of patients with and without the recurrence of AF.

Variables	PAF			non-PAF		
	No Recurrence	AF Recurrence	P Value	No Recurrence	AF Recurrence	P Value
Patients, n (%)	50	23		26	19	
Male sex, n (%)	42(84)	19(82.6)	1	22(84.6)	19(100)	0.13
Age, years (IQR)	57.0(49.0–62.0)	59.0(54.5–62.0)	0.56	58.5(53.5–62)	56.0(48.5–62)	0.39
BMI, kg/m^2^ (IQR)	24.3(22.1–27.9)	22.3(21.2–24.4)	0.015*	23.7(21.4–26.1)	24.5(21.4–26.1)	0.68
Duration of AF, months (IQR)	31.0(10.5–72.0)	36.0(4.5–60.0)	0.42	15.5(5.3–36.0)	36.0(20.5–72.0)	0.008*
Hypertension, n (%)	17(34)	6(26.1)	0.594	5(19.2)	9(47.4)	0.057
Diabetes mellitus, n (%)	4(8)	3(13.0)	0.671	1(3.8)	6(15.8)	0.295
Stroke, n (%)	2(4)	3(13.0)	0.317	2(7.7)	3(15.8)	0.636
History of TCM, n (%)	4(8)	1(4.3)	1	1(3.8)	5(15.8)	0.295
Pre-ablation anti-arrhythmic drugs						
Class Ⅰ AAD, n (%)	26(52)	11(47.8)	0.804	7(27.0)	2(10.5)	0.264
Class Ⅲ AAD, n (%)	1(2.0)	0(0.0)	1	1(3.8)	0(0.0)	1
βblocker, n (%)	20(40)	7(30.4)	0.602	12(46.1)	11(57.9)	0.55
ACEI/ARB, n (%)	15(30)	3(13.0)	0.151	7(26.9)	9(47.4)	0.212
Statin, n (%)	9(18)	3(13.0)	0.74	6(23.1)	8(36.8)	0.34
LV ejection fraction, % (IQR)	69.0(65.0–72.0)	67.0(65.0–70.0)	0.38	63.5(60.0–65.8)	64.0(54.5–68.0)	0.94
LVDd mm (IQR)	50.0(45.3–51.8)	48.0(46.0–52.5)	0.97	48.0(45.0–50.8)	48.0(46.5–52.0)	0.55
Left atrial anteroposterior echocardiographic dimension, mm (IQR)	40.0(36.0–42.8)	40.0(36.0–42.5)	1	42(37.3–45.5)	41(39.5–46.0)	0.49
NT proBNP, pg/ml (IQR)	60.5(24.8–146.0)	70.0(54.8–108.5)	0.35	422(322–671)	375(190–928)	0.99

Data are n (%), and median (IQR; 25%―75% interquartile range).

* significant difference

AAD, anti-arrhythmic drug; ACEI, angiotensin converting enzyme inhibitor; AF, atrial fibrillation; ARB, angiotensin II receptor blocker; BMI, body mass index; LV, left ventricle; LVDd, left ventricular end-diastolic diameter; non-PAF, non-paroxysmal atrial fibrillation; PAF, paroxysmal atrial fibrillation; TCM, tachycardia-induced cardiomyopathy

The number of people with PV variation was not statistically different among the 4 groups (Table 5).

**Table 5:** PV volume and LA volume with and without the recurrence of AF.

Variables	PAF			non-PAF		
	No Recurrence	AF Recurrence	P Value	No Recurrence	AF Recurrence	P Value
Patients, n	50	23		26	19	
Total PV volume, cm^3^/BSA(m^2^)	10.6 ± 2.8	13.1 ± 3.9	0.013*	12.8 ± 3.1	11.5 ± 3.6	0.782
LA volume, cm^3^/BSA(m^2^)	44.8 ± 11.4	48.9 ± 11.5	0.330	58.3 ± 13.8	58.5 ± 15.7	0.952
Total PV+LA volume, cm^3^/BSA(m^2^)	55.5 ± 12.3	61.9 ± 14.1	0.181	71.1 ± 14.7	67.0 ± 18.4	0.972
Total PV volume / LA volume	0.249 ± 0.088	0.271 ± 0.066	0.321	0.229 ± 0.069	0.200 ± 0.048	0.512
Anatomical variation of PV						
Left PV variation (common trunk)	11(22.0)	6(26.1)	0.769	7(26.9)	5(26.3)	0.746
Accessary vein	10(20.0)	7(30.4)	0.378	9(34.6)	5(26.3)	1.00

Data are mean ± SD, and n (%).

* significant difference

AF, atrial fibrillation; BSA, body surface area; LA, left atrium; non-PAF, non-paroxysmal atrial fibrillation; PAF, paroxysmal atrial fibrillation; PV, pulmonary vein

Given the anatomical variation of PV, especially PV common trunk, and technical difficulty in determining PV ostium, PV ostial area and LA diameter were obtained in 63 patients (Table 6).

**Table 6:** PV ostial area and LA diameter.

Variables	PAF			non-PAF		
	No Recurrence	AF Recurrence	P Value	No Recurrence	AF Recurrence	P Value
Patients, n	28	12		15	8	
RSPV area, cm^2^/BSA(m^2^)	1.02 ± 0.31	1.27 ± 0.24	0.104	1.08 ± 0.34	0.97 ± 0.34	0.835
RIPV area, cm^2^/BSA(m^2^)	0.87 ± 0.27	1.13 ± 0.30	0.039*	0.94 ± 0.29	0.95 ± 0.25	0.999
LSPV area, cm^2^/BSA(m^2^)	1.16 ± 0.48	1.33 ± 0.41	0.544	1.11 ± 0.38	1.14 ± 0.40	0.988
LIPV area, cm^2^/BSA(m^2^)	0.71 ± 0.23	0.74 ± 0.19	0.976	0.69 ± 0.31	0.66 ± 0.12	0.988
Two RPVs area, cm^2^/BSA(m^2^)	1.89 ± 0.49	2.40 ± 0.46	0.042*	2.02 ± 0.53	1.92 ± 0.52	0.980
Two LPVs area, cm^2^/BSA(m^2^)	1.87 ± 0.60	2.07 ± 0.38	0.382	1.80 ± 0.54	1.80 ± 0.46	0.999
Two SPVs area, cm^2^/BSA(m^2^)	2.18 ± 0.72	2.60 ± 0.59	0.104	2.19 ± 0.64	2.11 ± 0.70	0.997
Two IPVs area, cm^2^/BSA(m^2^)	1.58 ± 0.40	1.87 ± 0.39	0.145	1.64 ± 0.38	1.61 ± 0.31	0.999
Four PVs area, cm^2^/BSA(m^2^)	3.76 ± 0.98	4.47 ± 0.75	0.022*	3.82 ± 0.89	3.72 ± 0.87	0.794
LA diameter						
LA longitudinal, mm/BSA(m^2^)	33.8 ± 4.8	34.3 ± 3.3	0.996	33.5 ± 5.2	35.2 ± 4.6	0.917
LA anteroposterior, mm/BSA(m^2^)	18.3 ± 3.2	19.3 ± 4.5	0.995	19.0 ± 4.5	18.1 ± 3.2	0.994
LA top―mitral annulus, mm/BSA(m^2^)	28.4 ± 4.1	29.8 ± 3.1	0.712	30.7 ± 4.3	29.8 ± 2.9	0.955
LA transverse, mm/BSA(m^2^)	29.6 ± 3.5	28.8 ± 3.6	0.971	31.8 ± 4.1	34.3 ± 6.7	0.955

Data are mean ± SD.

* significant difference

AF, atrial fibrillation; BSA, body surface area; Four PVs, RSPV+RIPV+LSPV+LIPV; LA, left atrium; LIPV, left inferior PV; LSPV, left superior PV; non-PAF, non-paroxysmal atrial fibrillation; PAF, paroxysmal atrial fibrillation; PV, pulmonary vein; RIPV, right inferior PV; RSPV, right superior PV; Two IPVs, RIPV+LIPV; Two LPVs, LSPV+LIPV; Two RPVs, RSPV+RIPV; Two SPVs, RSPV+LSPV

**Fig 4:**
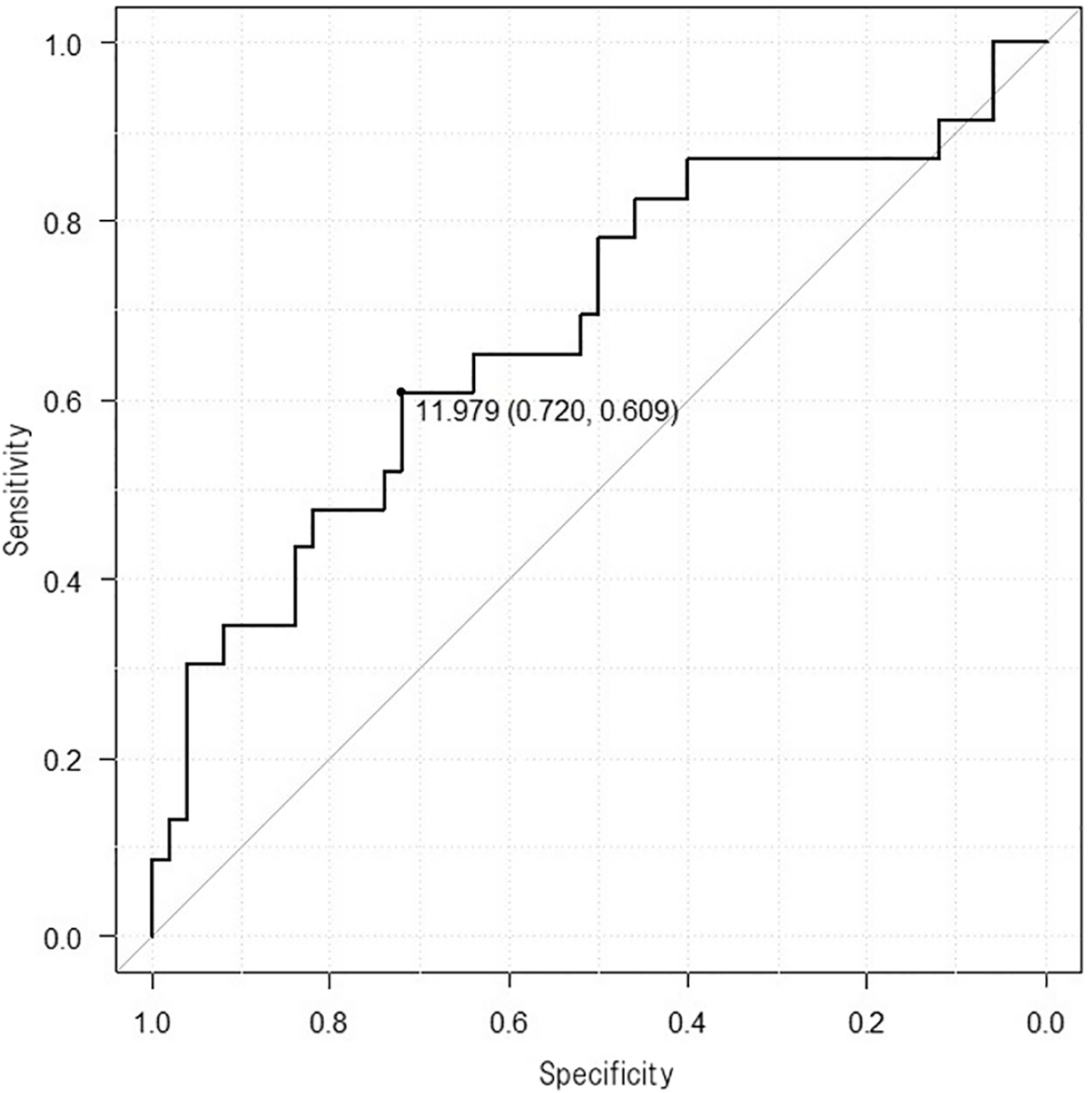
Receiver-operating characteristics curve. Success after ablation of atrial fibrillation (AF) for pulmonary vein volume below 12.0 cm^3^ / BSA (m^2^) in the paroxysmal AF group.

In the PAF group, total PV volume, area of right inferior PV, area of right superior and right inferior PVs, and area of the four PVs were independent predictors of AF recurrence. Difference was not observed in LA dimensions or LA volume between patients with and without AF recurrence. PV variations including accessory veins were equally distributed among the different outcomes of RFCA. Logistic regression analysis showed that the probability of AF recurrence was higher in patients with total PV volume greater than 12.0 cm^3^/BSA (m^2^) (AUC 0.682, 95%CI 0.541―0.822), area of right inferior PV greater than 0.844 cm^2^/BSA (m^2^) (AUC 0.768, 95%CI 0.617―0.919), area of right PVs greater than 1.985 cm^2^/BSA (m^2^) (AUC 0.765, 95%CI 0.613―0.916), and area of four PVs greater than 3.656 cm^2^/BSA (m^2^) (AUC 0.759, 95%CI 0.607―0.911) (Fig 4).

In the non-PAF group, no independent risk factor of either LA or PV parameters was found for the postoperative recurrence after RFCA (Tables 5 and 6).

Comparing all PAF patients with all non-PAF ones, LA transverse diameter and LA volume were greater in the non-PAF group than those in the PAF group (P< 0.05 and P< 0.01, respectively). However, no difference in PV volume or other LA diameters was observed between the PAF and non-PAF groups (Table 7).

**Table 7:** PV volume and LA dimension in the PAF and non-PAF groups.

Variables	PAF		non-PAF		
	Patients, n		Patients, n		P Value
Total PV volume, cm^3^/BSA(m^2^)	73	11.4 ± 3.4	45	12.2 ± 3.4	0.127
LA volume, cm^3^/BSAm^2^	73	46.1 ± 11.6	45	58.4 ± 14.6	<0.001*
Total PV+LA volume, cm^3^/BSA(m^2^)	73	57.5 ± 13.3	45	70.6 ± 16.3	<0.001*
Total PV volume / LA volume	73	0.256 ± 0.082	45	0.217 ± 0.063	0.010*
LA diameter					
LA longitudinal, mm/BSA(m^2^)	40	33.9 ± 4.4	23	34.1 ± 5.0	0.909
LA anteroposterior, mm/BSA(m^2^)	40	18.6 ± 3.6	23	18.7 ± 4.1	0.915
LA top―mitral annulus, mm/BSA(m^2^)	40	28.9 ± 3.9	23	30.4 ± 3.9	0.148
LA transverse, mm/BSA(m^2^)	40	29.4 ± 3.6	23	32.7 ± 5.3	0.020*

Data are mean ± SD.

* significant difference

BSA, body surface area; LA, left atrium; non-PAF, non-paroxysmal atrial fibrillation; PAF, paroxysmal atrial fibrillation; PV, pulmonary vein

Total patients were divided into two groups, namely, patients with and without recurrence of AF. Significant difference was not found in total PV volume or LA volume between these groups (Table 8).

**Table 8:** PV volume and LA volume in the non-recurrent and recurrent groups.

Variables	No Recurrence	AF Recurrence	P Value
Patients, n	76	42	
Total PV volume, cm^3^/BSA(m^2^)	11.4 ± 3.1	12.3 ± 3.9	0.151
LA volume, cm^3^/BSAm^2^	49.4 ± 13.9	53.2 ± 14.6	0.126
Total PV+LA volume, cm^3^/BSA(m^2^)	60.8 ± 15.2	65.6 ± 16.9	0.106
Total PV volume / LA volume	0.242 ± 0.083	0.239 ± 0.069	0.888

Data are mean ± SD. AF, atrial fibrillation; BSA, body surface area; LA, left atrium; PV, pulmonary vein

## Discussion

In AF patients, whether paroxysmal or persistent, successful maintenance of sinus rhythm has been reported to relieve symptoms, improve exercise capacity, and have potential advantages in reduction of mortality risk [7,8,9]. Moreover, because AF itself may induce electrical and structural remodelings to promote the formation of a substrate for sustained AF [10], maintaining sinus rhythm is an important therapeutic interim goal to prevent disease progression.

The combination of catheter ablation and antiarrhythmic drug (AADs) therapy is a fundamental approach for rhythm control strategy. AADs are the first-line therapy for AF patients, in whom class I and III AADs and beta-blockers significantly lowered the recurrence rate of AF in the meta-analysis studies. As long-term use of class III drugs is still considered to have cardioversion effects in persistent AF patients, amiodarone may be a most effective agent in spite of its various side effects [11,12,13]. Meanwhile, in this study, we mainly administered bepridil, known as a non-selective Ca^2+^, Na^+^, and K^+^ channel blocker, as this agent may have more powerful effects than amiodarone in achieving and maintaining sinus rhythm even among persistent AF patients [14], although this drug is not internationally approved for AF treatment. Some reports were found concerning the relationship between Ca^2+^, Na^+^, and K^+^ channels and the conditions in which AF is likely to be initiated or maintained. Downregulation of inward L-type Ca^2+^ current and enhanced outward K^+^ current induce shortening of atrial action potential duration, thereby sustaining AF. Furthermore, the cellular Ca^2+^ overload produced by AF-induced tachycardia potentially causes enhanced diastolic Ca^2+^ leak, which may increase ectopic premature beats and trigger AF initiation. By contrast, discrepant findings have been reported on Na^+^ channel remodeling in AF [15,16,17,18,19], where AF becomes more resistant to pharmacological cardioversion by class I drugs along with AF prolongation [11,12].

Catheter ablation is becoming increasingly common, but its results vary widely. In the recent largest prospective Catheter Ablation vs. Antiarrhythmic Drug Therapy for AF (CABANA) trial expecting a long-term benefit of ablation in the composite endpoint, including total mortality, in comparison with medication, catheter ablation for AF is not necessarily superior to drugs for reducing major outcomes. Therefore, we now have to select patients who would have potential to gain a therapeutic benefit from ablation that outweighs the preprocedural risk of AF.

PV isolation has been reported to be superior to antiarrhythmic medications at reducing AF burden especially in younger PAF patients without underlying structural heart disease [20,21]. As the younger PAF patients have less LA remodeling, successful blockade of AF initiation trigger works effectively. Considering that LA remodeling is related to LA enlargement, we investigated the preprocedural factors in selecting younger AF patients for ablation by using CT imaging in this study.

Considerable variations exist in PV anatomy and anatomical alterations of PVs, including common ostia and additional veins detected in 18―45% of AF patients [22]. Although several reports demonstrated a higher AF recurrence rate in anatomical variants, others showed an increased incidence of AF recurrence in normal anatomical PV compared with variant anatomy, or they did not find a relation between PV anatomy and AF recurrence [23,24,25]. In the present study, multivariate regression analysis did not indicate that PV variation was an independent risk factor for postoperative recurrence (Table 5).

The size of PV and LA is considered to be a relevant risk factor for AF, postoperative recurrence and progression of PAF to non-PAF. Previous reports demonstrated that PAF patients show a greater longitudinal dimension of LA and a larger superior PV volume than normal control subjects with normal sinus rhythm [22,26,27,28]. This finding suggests that the larger size of PV and LA is a causative factor of AF or a result from an increment of LA pressure produced by PAF. However, because the risk factor for postoperative recurrence may not be the same to that for AF and progressing PAF to non-PAF, we evaluated whether LA and PV size provided by MDCT may predict the success rate of RFCA involving PV isolation procedure for AF.

### Influence of PV and LA size on AF recurrence after RFCA in PAF patients

We demonstrated that greater total PV volume and PV ostial area in PAF patients were related to AF recurrence after RFCA. Given the considerable PV anatomical variations, we employed 3D reconstructed models from the MDCT images and performed volume measurement in the four PVs automatically by using a specialized software. We found a cut-off value of 12.0 cm^3^/BSA (m^2^) for the total PV volume, below which there was a good predictive value for sinus rhythm maintenance after RFCA in the PAF group.

**Fig 5:**
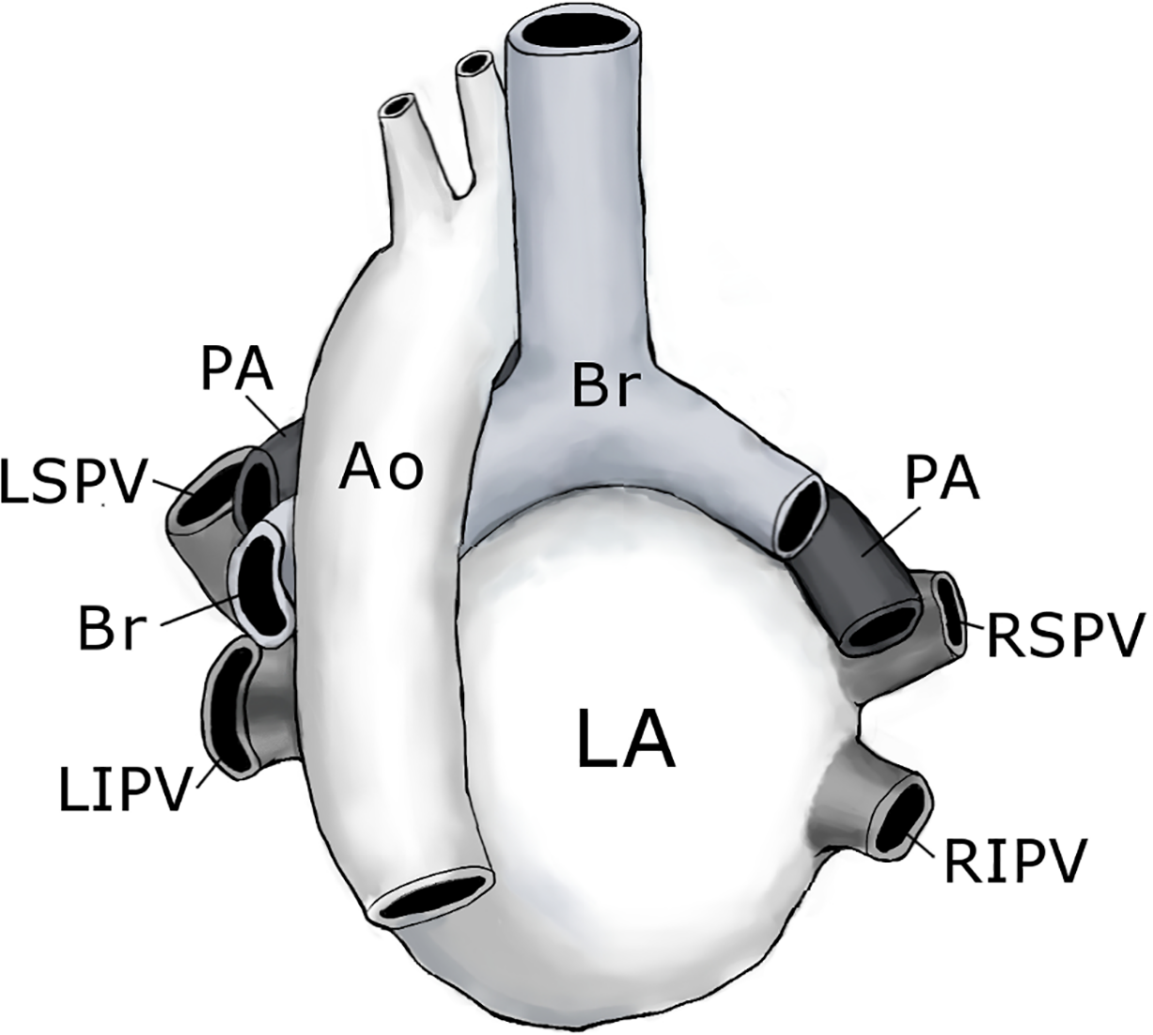
Heart and great vessels, hardened in situ, posterior aspect. The superior Pulmonary veins (PVs) enter the left atrium (LA) from a superior, anterior, and lateral direction and the inferior PVs from the posterior direction. The right and left pulmonary arteries (PAs) lie just above and parallel to the right and left superior PVs. The ostium of the left inferior PV lies between the LA and descending aorta (Ao). There is neither artery nor bronchi (Br) adjacent to the right inferior PV. LSPV, left superior PV; LIPV. left inferior PV; RSPV, right superior PV; RIPV. right inferior PV.

We also measured both superoinferior and anteroposterior lengths of each PV ostium using the 3D reconstructed models from the MDCT images and calculated each PV ostial area. In terms of the increase in PV ostial area, the right inferior PV was the most pronounced in this study. At present, only a few studies have directly compared single PV ostial diameter using CT and confirmed enlargement of superior PVs as an independent risk factor for AF recurrence following RFCA [29], which was inconsistent with our data. PV ostia are considered to have an elliptical shape with a long axis in vertical orientation [30]. Thus, calculation of PV ostial areas in this study may be more accurate to evaluate changes in anatomical PV size compared with single PV ostial diameter measurements. Furthermore, the anatomy of LA, PVs and adjacent organs may indicate a marked enlargement of the right inferior PV ostium as shown in this study (Fig 5). Although both superior PVs are just under the pulmonary arteries and left inferior PV lies astride the descending aorta, the right inferior PV lacks adjoining organs, becoming easily dilated depending on the increase in PV pressure. In particular, the left inferior PV straddles and sometimes stretches over the descending aorta, often resulting in an impression on the dorsal side of its PV and flattening dorsoventrally. Therefore, the left inferior PV had a smaller ostial ratio of anteroposterior /superoinferior length than the right superior, right inferior, and left superior PVs (0.622 ± 0.197 vs 0.783 ± 0.194, 0.771 ± 0.126 and 0.823 ± 0.186, P< 0.001).

We should emphasize that people spend two-thirds of daily life activities in the upright posture. Thus, pulmonary circulation with low perfusion pressure is sensitive to changes in posture with its distribution of flow and pressure above and below the heart [31]. In the upright posture, inferior PVs seem much more important than superior PVs as a risk factor for AF recurrence because intra LA pressure becomes increasingly positive as the height decreases. There is approximately 6 cmH_2_O in hydrostatic pressure difference between superior PVs and inferior ones because of the top―mitral LA distance of 6 cm. Furthermore, in the upright posture, when alveolar pressure equals to the surrounding tissue pressure, the pulmonary capillaries are constant from the top to the bottom of the lung. Both arterial and venous pressures change in proportion to the distance above or below the heart. Thus, in the upper lung, when arterial pressure approaches or falls below capillary pressure, pulmonary flow decreases or stops. Conversely, in the lower lung, when arterial and venous pressures surpass capillary pressure, flow is not inhibited by capillary pressure but maintained. Thus, the dynamic pressure calculated as half the density times the velocity squared becomes higher in the lower PVs than in the upper PVs. Because venous transmural pressure is the sum of the hydrostatic and dynamic pressures, inferior PV transmural pressure becomes higher than that of superior PVs by more than 6 cmH_2_O of hydrostatic pressure difference in the upright position. Therefore, inferior PVs are considered as being more influential than superior PVs as a risk factor for AF recurrence because an increment of intra-atrial pressure accelerates the rate and organization of waves emanating from PVs underlying stretch-related AF. In particular, elevating intra-atrial pressure above 10 cmH_2_O makes the LA―PV junction the source of dominant rotors [32]. Acute mechanical stretch-induced enhancement of the automaticity of the pulmonary vein myocardium through the opening of the stretch-activated channels is revealed in the extracted heart from a guinea pig [33]. Although PV mechanical stretch associated with postural change is gentler and has more chronic burden, the inferior PVs automaticity might serve as a trigger for the initiation and maintenance of AF.

We mentioned that hemodynamics should differ depending on the posture, suggesting the possibility of AF attack during daytime, although we have no data on the onset time of AF attacks. Earlier studies have shown a degree of daily variation in the onset of AF, possibly due to variations in the tonus of the autonomic nervous system. Attacks are more common in the morning and at night [34], but higher frequency has also been reported during daytime [35].

### Influence of PV and LA size on AF recurrence after RFCA in non-PAF patients

We found that AF duration is an independent risk factor for postoperative recurrence in the non-PAF group. Moreover, all non-PAF patients showed significantly greater LA volume and LA transverse length compared with all PAF ones. However, LA size did not have any effect on the outcome in non-PAF patients.

Hof et al. [25] reported LA volume as an independent predictor of AF recurrence. Zhuang et al. [36] performed a systemic review and meta-analysis to demonstrate the association between LA diameter and AF recurrence after single circumferential PV isolation, whereas other meta-analyses denied this possibility [37]. A notable limitation of evaluating LA size in these previous meta-analyses is the fact that dilated LA induced by AF may modify the ellipsoidal shape into a more trapezoidal shape because of atrialization of PV [38]. Thus, a simple linear dimension may not be representative of LA size [39,40], such as anteroposterior diameter measured by the end-systolic LA in the echocardiographic parasternal long axis view. With LA geometry and changes in shape, magnetic resonance and CT could be more appropriate methods for evaluation of LA size. One report revealed that LA volume using magnetic resonance angiographic imaging was not related to recurrence of AF after ablation, which is consistent with our data [41].

### Possible mechanism of AF recurrence after RFCA in the PAF and non-PAF groups

In PAF patients, greater total PV volume and PV ostial area were related to AF recurrence after RFCA. The PVs are preferred sites for reentrant arrhythmias [42,43] in clinical status, where AF causes an increase in LA pressure and concomitant PV activation because of shorter refractory periods inside the PVs and produces easy induction of PV reentry with premature stimulation from the PVs. In a canine model, compared with LA, PVs have shallow resting membrane potential associated with reduced inward-rectifier K^+^ current and shorter action potential duration associated with increased slow and rapid delayed rectifier K^+^ currents and reduced L-type Ca^+^ current. Such properties cause electrical instability and create a vulnerable substrate that may contribute to conduction delays and the promotion of re-entry in PVs [44]. In addition, both enhanced Ca^2+^ transient and increased Na^+^/Ca^2+^ exchanges are considered a mechanism to cause focal ectopic activity that triggers AF [45].

Since the PV wall is thinner and more compliant than that of LA, even a small increment of LA pressure may initially lead to significant enlargement of PV ostia accompanied with increase in PV volume, which may adversely affect the success rate of the procedure in the PAF group. On the other hand, all non-PAF patients exhibited significantly greater LA volume and LA transverse length compared with all PAF ones. Therefore, PAF patients who benefit from PV isolation are supposed to have only PV localized problems and those who have recurrent AF might have potential remodeling extending to LA even though there was still no obvious LA volume enlargement. However, PV volume was not statistically different between the PAF and non-PAF groups. Our data suggest that PV volume increases from patients without recurrence to those with recurrence in PAF. It reaches the maximum before AF becomes persistent in progression to non-PAF. By contrast, continuously elevated LA pressure further increases LA dimension transversely, resulting in enlargement of LA in non-PAF patients. This finding suggests that LA wall stress calculated as the pressure times radius divided by wall thickness becomes higher in non-PAF patients than in PAF patients. However, PV wall stress remains unchanged under constant pressure and wall thickness. With longer AF duration, significant structural and electrical remodeling would have occurred due to repeated stimulations of LA and PV [36]. Furthermore, dominant-frequency analysis points to an evaluation of mechanisms in AF patients, with PV sources becoming less predominant as AF becomes more persistent and atrial remodeling progresses. This would change the sensitivity of atrial muscles to RFCA energy and adversely affects the success rate of the operation.

### Limitations

The limitation of this study is the absence of defined criteria for the measurement of the PV ostial volume due to the difficulties of the assessment of the distal point of PV. Previous investigators proposed the midpoint of the PV 1 cm of the ostium as the distal point [22] because myocardial muscle fibers are generally agreed to extend from the LA into all the PVs for 1 to 3 cm. This sleeve length was not changed in AF patients [46]. In this area, the thickness of the muscular sleeve is the highest at the proximal ends and gradually decreases distally [4,47]. PV focal firing may trigger AF or act as a rapid driver to maintain arrhythmia. Thus, we used the simultaneously acquired voltage map and defined the PV distal border as the first major branch referred to the main LA activation located inside the main trunk of PV.

We did not use contact force ablation catheters. Therefore, this might have affected our slightly higher recurrence rate in PAF patients [48]. We have no data on the electrophysiological parameters in the PVs and ostium, or reconnection rate in AF-recurrent patients during the second ablation session, and we did not follow up the patients by using contrast enhance CT. Therefore, the possible reasons for the recurrence and relationship to PV/LA anatomy and volume change after ablation were unclear in this study.

## Conclusions

We found that total PV volume was significantly larger in PAF patients with recurrent AF than in those without recurrence after RFCA. This finding suggests that potential electrical remodeling of LA and enlargement of PV precede anatomical change of LA in PAF patients.
